# Deconvolution and Analysis of the ^1^H NMR Spectra of Crude Reaction Mixtures

**DOI:** 10.1021/acs.jcim.3c01864

**Published:** 2024-04-04

**Authors:** Maxwell C. Venetos, Masha Elkin, Connor Delaney, John F. Hartwig, Kristin A. Persson

**Affiliations:** † Department of Materials Science and Engineering, University of California, Berkeley, California 94720, United States; ‡ Department of Chemistry, University of California, Berkeley, California 94720, United States; § Molecular Foundry, Lawrence Berkeley National Laboratory, Berkeley, California 94720, United States

## Abstract

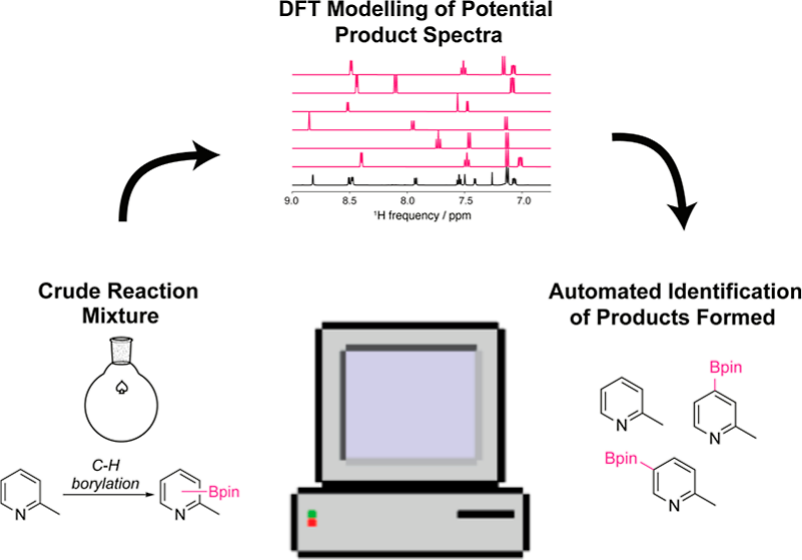

Nuclear magnetic resonance (NMR) spectroscopy is an important analytical technique in synthetic organic chemistry, but its integration into high-throughput experimentation workflows has been limited by the necessity of manually analyzing the NMR spectra of new chemical entities. Current efforts to automate the analysis of NMR spectra rely on comparisons to databases of reported spectra for known compounds and, therefore, are incompatible with the exploration of new chemical space. By reframing the NMR spectrum of a reaction mixture as a joint probability distribution, we have used Hamiltonian Monte Carlo Markov Chain and density functional theory to fit the predicted NMR spectra to those of crude reaction mixtures. This approach enables the deconvolution and analysis of the spectra of mixtures of compounds without relying on reported spectra. The utility of our approach to analyze crude reaction mixtures is demonstrated with the experimental spectra of reactions that generate a mixture of isomers, such as Wittig olefination and C–H functionalization reactions. The correct identification of compounds in a reaction mixture and their relative concentrations is achieved with a mean absolute error as low as 1%.

## Introduction

The synthesis of novel chemical compounds is a crucial component of organic chemistry, and the preparation of novel compounds requires significant experimentation to identify reaction conditions that form products with acceptable yield, chemo- and stereoselectivity, purity, cost, and environmental footprint. High-throughput experimentation (HTE) techniques allow researchers to conduct hundreds or thousands of experiments quickly, but the analysis of those experiments remains a significant bottleneck.

The most common approach to identifying and quantifying the reaction products in reaction mixtures in a high-throughput fashion is gas or liquid chromatography. This approach requires authentic standards of the expected products to confirm the presence of reaction products and calibration curves to accurately quantify product concentrations.^1^ When only a small number of products are being considered, it is manageable to isolate or independently generate an authentic standard for each potential product or byproduct. However, if a large library of novel compounds is made, isolating or independently synthesizing each compound under consideration is prohibitively time-consuming. The synthesis of large libraries of compounds that span unexplored areas of chemical space is critical for the diversity-oriented synthesis approach in drug discovery,^2^ the generation of training data for machine learning models,^3,4^ and the automated discovery of novel reactions.^5^ Methods for reaction analysis that are based on mass spectrometry have limited ability to differentiate compounds that are isomers of one another and cannot be used easily to quantify the concentrations of novel products.^6−8^

In contrast to gas or liquid chromatography, nuclear magnetic resonance (NMR) spectroscopy is commonly used to determine the relative concentrations of reaction products without the need for an authentic standard or calibration curve. Moreover, NMR spectroscopy provides detailed information about the identity of products, allowing it to be used to determine the structure of new chemical compounds. The use of NMR spectroscopy to analyze the data generated by HTE would dramatically expand the capabilities of HTE, especially when large numbers of novel compounds are synthesized. However, in most cases, NMR spectra are analyzed by experts, slowing down the process of an otherwise automated workflow. A tool that automatically analyzes the NMR spectra of an unpurified reaction mixture (henceforth termed the crude spectrum) would greatly enable HTE campaigns.

Prior approaches to the automated analysis of NMR spectra employed machine learning models to analyze the spectra. The majority of current models analyze the spectrum of a pure sample of an unknown compound to determine its identity.^9−13^ These models can be employed only on spectra that contain a single compound, but full workflows capable of analyzing multicomponent spectra are becoming more commonplace.^14−18^ Although powerful for their trained tasks, these machine-learning models require a database containing spectra of each potential component and cannot identify novel compounds.

Markov Chain Monte Carlo (MCMC) methods have been used for a diverse range of applications related to NMR spectroscopy,^19−23^ including MCMC methods for the deconvolution and quantification of multicomponent spectra, given a library of known compounds.^24,25^ Like the machine learning approaches, this approach has not been used to identify or quantify compounds for which a spectrum is not already documented, and this limitation leads to the same need for authentic standards that hampers analysis by gas and liquid chromatography. Therefore, a tool is needed that performs automated analysis of crude NMR spectra without the NMR spectra of pure individual components.

Hamiltonian Monte Carlo Markov Chain (HMCMC) modeling is a statistical sampling method that allows for the efficient sampling of a conditional probability distribution when only a joint probability distribution is available. By reframing the NMR spectrum of a reaction mixture as a joint probability distribution, it could be possible to use HMCMC to fit spectra predicted by density functional theory (DFT) to crude reaction spectra. In most cases, a synthetic chemist knows which products or side products are likely to have formed in a reaction and can provide structures of all relevant products. It is also becoming more common for reaction-prediction models to enumerate probable reaction products.^26−31^ One could imagine creating a tool that considers a list of products provided by a user, identifies which products are in the crude spectrum, and determines their relative concentrations. This approach relies on the use of computed spectra in place of experimentally determined spectra of authentic products, thereby dramatically increasing its utility by enabling the identification and quantification of products in a reaction mixture that have not been previously described.

To address this need, we developed a combined DFT–HMCMC workflow to analyze crude NMR spectra of reaction mixtures without the need for an experimentally generated library of known spectra. This workflow consists of: (1) obtaining ground state conformers of a set of candidate compounds, (2) calculating the NMR isotropic shielding constants via DFT to predict the ^1^H NMR spectrum of each compound in solution, and (3) varying the stoichiometric weights and chemical shifts of each candidate compound via HMCMC analysis to identify the products and the relative ratios of those products. We show that this model can analyze experimental spectra of various reaction types, enabling the automatic identification of reaction components and the quantification of their relative concentrations.

## Methods

### NMR Simulations

All molecular dynamics and ab initio calculations were performed at the National Energy Research Scientific Computing (NERSC) facility Cray XC40 computer running an Intel Xeon Processor E5–2698 v3 node with 128 GB of memory. For each compound, a conformer search was performed using the Conformer-Rotamer Ensemble Sampling Tool (CREST),^32^ simulating a solvent environment of CHCl_3_. The ground state conformer found via CREST was optimized, and the NMR shielding tensors and *J*-coupling tensors were calculated using approximate density functional theory (DFT) in QChem v6.0.1.^33^ A generalized gradient approximation (GGA) density functional was used following an implementation of Becke’s *B3LYP* GGA functional.^34^

Dunning’s correlation consistent triple-ζ cc-PVTZ basis set^35^ was used to optimize the geometry of the structure and calculate the shielding tensor, as suggested by Flaig et al.^36^ Jensen’s polarization-consistent pcJ-2 basis set^37^ was used for the *J*-coupling calculation. These functionals and basis sets were chosen to balance speed and accuracy; while more accurate methods exist to calculate isotropic shieldings and *J*-couplings,^37−43^ many of these methods are prohibitively expensive for application in high-throughput workflows. We used a linear scaling approach to compensate for the systematic errors in DFT calculations. We show that the fitting procedure developed herein enables the accurate identification of products despite the relatively low accuracy of the DFT calculations.

**Figure 1:**
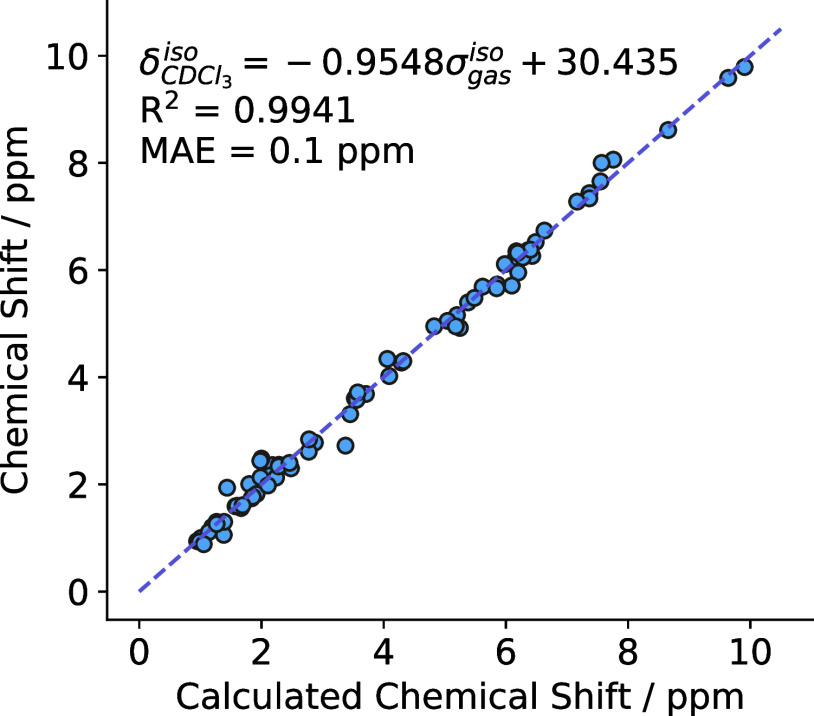
Experimental isotropic chemical shielding versus the calculated isotropic chemical shift derived from the DFT-calculated isotropic nuclear shielding using the equation δ^iso^ = −0.9548σ_gas_^iso^ + 30.435, which has an *R*^2^ = 0.9941 and an MAE of 0.1 ppm.

The HMCMC analysis is robust against small (ca. 0.1 ppm) errors in isotropic chemical shifts, but it cannot accommodate large errors in the predicted chemical shifts. The accuracy of the isotropic shifts obtained from DFT calculations was improved by correlating the calculated isotropic shieldings to the experimentally observed isotropic shifts rather than referencing the calculated chemical shifts to the calculated chemical shift of a standard compound, such as trimethylsilane (TMS). Kwan and Liu^43^ have shown that such calibrations serve as simple rovibrational corrections to the predicted isotropic shifts of the ground state conformations of small molecules. To create a calibration line, 33 experimental NMR spectra of compounds in CDCl_3_ solvent were obtained from the Spectral Database for Organic Compounds (SDBS).^44^ The selected compounds were subjected to the procedure above to obtain isotropic shielding for nonlabile protons. The error in the calibration curve shown in Figure 1 is 0.1 ppm and fits a functional form of
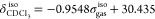
1in which  is the isotropic chemical shift in a CDCl_3_ solvent environment, and σ_gas_^iso^ is the DFT-calculated isotropic nuclear shielding. This relationship was used to convert the nuclear shieldings calculated by DFT to isotropic chemical shifts.

### Spectral Fitting with HMCMC

In our method, an initial trial spectrum is generated as a linear combination of the spectra calculated for the compounds hypothesized to be present in the reaction mixture. That trial spectrum is then fit to the experimentally observed NMR spectrum. To do so, we reframe the NMR spectrum as a statistical distribution of stoichiometric coefficients (relative concentrations) and NMR parameters (chemical shifts) and use Hamiltonian Markov Chain Monte Carlo (HMCMC) to fit a trial spectrum to the observed spectrum. HMCMC is a technique that is widely used to sample from target distributions when direct sampling is not available. Typical MCMC methods are inefficient for this application because they scale poorly with the number of dimensions. HMCMC uses the principles of Hamiltonian dynamics to produce Markov chains and scales more efficiently^45^ than MCMC methods as the number of dimensions increases.

**Figure 2:**
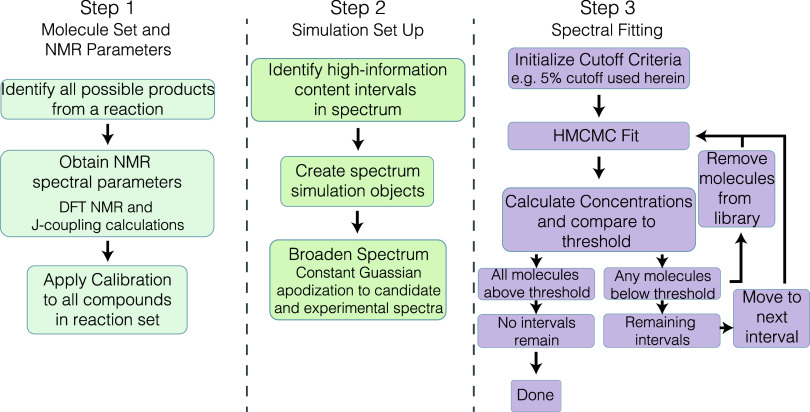
Overview of the three-step procedure for deconvoluting NMR spectra.

A workflow was created around HMCMC modeling, as shown in Figure 2, to determine the true composition of an NMR spectrum containing multiple species, given a set of candidate products. In the first step, approximate NMR spectra for each candidate compound are calculated with DFT, and the calculated isotropic shielding is converted to isotropic shifts by using eq 1. Next, to simplify fitting the spectrum, regions of the spectrum that are most likely to eliminate potential products are identified and fit iteratively. The spectrum is divided into subspectra by creating intervals of ±0.5 ppm around each observed peak. Overlapping intervals are merged across all compounds in the spectral library, and a set of spectral intervals is determined. The information content (described in Spectral Information Content below) of these intervals is used to rank the order in which intervals are fit. Finally, iterative HMCMC fitting is used to remove compounds present in low concentrations based on a cutoff criterion.

To a first approximation, a spectrum in the time domain, *S*(*t*), may be modeled as a sum of time signals, *s*(*t*), for each proton, *l*
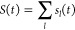
2in which
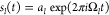
3*a*_*l*_ is the weight of the time signal, and Ω_*l*_ is the frequency for each proton. The presence of *J*-couplings splits the signals into a predictable pattern, wherein the number of splittings is given by the familiar *N* + *1* rule, with the spacing between split peaks given as *J*/2 and the new intensities following Pascal’s triangle
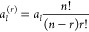
4

To approximate the decay characteristic of real NMR signals, an exponential decay term, λ, is added to give
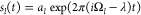
5

Upon Fourier transform into the frequency domain, the real part of the spectrum is given by a sum of Lorentzian lineshapes
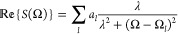
6

In the case of an experimentally acquired spectrum, the frequencies are referenced to some compound, often TMS, and chemical shifts, δ_*l*_, are used rather than the frequencies.

For a library of candidate compounds derived from chemical intuition, such as all possible constitutional isomers that could form by a C–H bond functionalization reaction, we calculate the conditional probability distribution of the component weighting factors, given the set of NMR parameters and the experimental spectrum, *S*^exp^
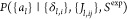
7

From the DFT simulations, however, all that is available is the joint probability distribution
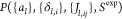
8

Fortunately, HMCMC allows one to generate samples from the conditional probability distribution given only the joint probability distribution (up to some constant). For more information on the HMCMC algorithm, we refer the reader to the numerous reviews on the topic.^46,47^

It is worth highlighting that our approach involves approximating the NMR spectrum as a probability distribution. This approximation introduces the potential for inaccuracies in the predicted chemical shifts, especially in spectral regions where significant overlap occurs. Nonetheless, the HMCMC algorithm accurately fits probability densities, leading to precise predictions of the concentration in our case. Moreover, leveraging the representation of the probability distribution of the NMR spectrum alongside the HMCMC technique accelerates convergence compared to conventional methods like least squares minimization.

All HMCMC runs were performed at the NERSC facility Cray XC40 computer running an Intel Xeon Processor E5–2698 v3 node with 128 GB of memory. The HMCMC runs followed the python package *NumPyro*(48,49) implementation, along with the No-U-Turn Sampler.^50^ Component weighting factors were sampled using a Half Cauchy prior distribution, and isotropic chemical shifts were sampled from a normal distribution centered around the isotropic shift with a standard deviation based on the error of the DFT calculations (0.1 ppm). The *J*-couplings were held constant during our procedure. While *J*-couplings and splitting patterns are useful for manual analysis of a spectrum, our procedure applies a broadening filter to the spectrum. This filter removes the fine detail from the splitting patterns and instead yields broadened Gaussian peaks with shapes dependent on the underlying splitting patterns and, to a much lesser extent, the frequencies of the *J*-couplings. As the *J*-couplings have a minimal effect on the lineshapes, we keep them constant. The HMCMC run was initialized with 1000 warmup samples and 3000 samples.

Calculated NMR lineshapes closely approximate delta functions, so a Gaussian apodization was applied to the simulated NMR lineshapes to broaden them and better approximate an experimental spectrum. Gaussian apodization was used because the resulting peak shape best matched the experimental peak shape, although our procedure can be used with any apodization method. A full width at half maximum (fwhm) of 2 Hz was determined (based on fitting spectra to the starting materials as described herein) and applied to each simulated NMR spectrum. To increase the gradient overlap between NMR peaks in the simulated and experimental spectra, an additional broadening filter was applied to both the experimental and simulated spectra. We found that a Gaussian apodization with a fwhm of 10 Hz was sufficient for increasing the gradient overlap in the HMCMC procedure. The HMCMC analysis required less than 3 h on a high-performance computing cluster in all cases studied. These HMCMC calculations were performed on dual-socket, 20-core, 2.1 GHz Intel Cascade Lake Xeon 6230 processors.

The HMCMC procedure was conducted iteratively with the library of candidate compounds to fit the simulated spectra to the experimental spectra. Compounds with a calculated concentration under a threshold level (initially set to 10%) were removed from the library, and HMCMC was repeated on the remaining candidates. This procedure was repeated until all remaining compounds were predicted to be present in the mixture, corresponding to the spectrum.

### Analytical Statistics

To measure the performance of our approach, we considered two goals: (1) correctly identifying which candidate compounds are present in a reaction mixture and (2) correctly calculating the relative concentrations of each compound in a reaction mixture. To determine the accuracy of the HMCMC procedure when finding the correct components of a mixture, we used classification accuracy. Classification accuracy is defined as the ratio of the correctly labeled components to all classifications
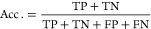
9in which TP is the number of true positives, TN is the number of true negatives, FP is the number of false positives, and FN is the number of false negatives.

We next calculated the accuracy of our approach that determines the concentrations of each compound in the mixture. Concentrations form a simplex (i.e., the sum of concentrations for the set is a constant, often normalized to 1), and statistics over the simplex do not follow the same rules as statistics over the unbound . Two common approaches for handling compositional data analysis over a simplex are the logratio analysis method and the unit simplex method; in this case, the unit simplex method was used for simplicity. For further information on compositional data analysis and these two approaches, we refer the reader to prior reports.^51,52^

Given a *D*-part composition, the composition vector is given as  in which each *x*_*i*_ is a composition. *Ĉ* is the closure operator, which normalizes the composition vector to 1 by dividing each element of the vector by the sum of the components. For the purposes of statistics over this simplex, the sample space, *S*^*D*^, is given as the set
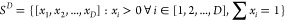
10

Given *N* repeated measurements (or samples) of the composition vector, we construct a *D* × *N* matrix, that contains observations of compositions , in which each  is a column vector of the *x*_*i*_ compositions over the observations. We are most interested in the measure of the central tendency *center*, ξ of the data, which is similar in interpretation to the mean of the data set in Euclidean statistics. The center of the data is given as
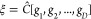
11in which *g*_*i*_ is the geometric mean of the component vector . The geometric mean is used instead of the arithmetic mean because the data are simplectic.^51,52^ In order to consider only a subset of the elements of a mixture, we form a subcomposition (or subsimplex). Given a *D*-part composition, a *C*-part subcomposition, for which *C* < *D*, may be formed via , in which all *x*_*i*_ ∈ *C*.

To calculate the mean absolute error (MAE), we use an approach similar to that used by Matviychuk et al.,^53^ in which MAE is calculated from mole fractions
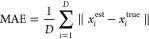
12

Here, the sum runs over the individual components of the *D*-component mixture, and *x*_*i*_^est^ represents the estimated mole fraction of component *i* as determined from the geometric mean of the HMCMC sample (as described in eq 11). The value *x*_*i*_^true^ is then the true mole fraction, as determined by the integrated NMR spectrum (as described below).

### Spectrum Acquisition

Simulated experimental spectra were obtained by combining known quantities of commercial reagents and directly acquiring an NMR spectrum. Crude experimental spectra were obtained by analyzing the reaction mixture at the end of an experiment without any purification. NMR spectra were acquired on 500 and 700 MHz Bruker instruments at the University of California, Berkeley NMR facility. Chemical shifts were reported relative to residual solvent peaks (CDCl_3_ = 7.26 ppm for ^1^H). Relative concentrations of components were determined by manual processing and integration of the NMR spectra.

The crude experimental spectra were obtained following either the borylation of arene C–H bonds or the olefination of aldehydes with a Wittig reagent. The borylation of C–H bonds was accomplished by the following procedure:^54^ In a nitrogen-filled glovebox, bis(pinacolato)diboron (B_2_Pin_2_, 10.4 mg, 0.04 mmol), (1,5-cyclooctadiene)(methoxy)iridium(I) dimer ([Ir(COD)OMe]_2_, 1.7 mg, 0.0025 mmol, 6.25 mol %), 3,4,7,8-tetramethyl-1,10-phenanthroline (Me_4_Phen, 1.2 mg, 0.005 mmol, 12.5 mol %), and tetrahydrofuran (THF, 1 mL, 0.04 M) were combined in a 4 mL vial equipped with a stir bar. The vial was heated at 80 °C for 1 h, and the catalyst mixture became dark red. To a separate vial was added substrate (0.203 mmol, 5 equiv). The catalyst mixture was added to the substrate, and the vial was sealed with a Teflon-lined cap. The reaction mixture was stirred at room temperature for 18 h. Volatile materials were evaporated with a rotary evaporator to obtain the crude products, which were directly analyzed by ^1^H NMR spectroscopy.

The olefination of aldehydes with a Wittig reagent was accomplished by the following procedure: To a solution of methyl (triphenylphosphoranylidene)acetate (100 mg, 0.30 mmol, 0.8 equiv) in water (5 mL, 0.08 M) was added benzaldehyde (40 mg, 0.38 mmol). The reaction mixture was heated at 80 °C for 1 h. The reaction was quenched by the addition of brine and extracted with ethyl acetate (EtOAc). The organic and aqueous layers were separated, the organic layer was dried with sodium sulfate (Na_2_SO_4_), and the solvent was evaporated with a rotary evaporator to obtain the crude products, which were directly analyzed by ^1^H NMR spectroscopy.

The starting materials and reagents used to assemble the reactions are known compounds, and their NMR spectra are either reported or can be immediately acquired. In such cases, we used reported or experimental spectra to refine the predicted spectra for these reaction components. To adjust the NMR parameters (isotropic shifts, *J*-couplings, and Gaussian fwhm broadenings), the simulated spectrum was fit to the experimental spectrum using the python package *Mrsimulator*.^55^*Mrsimulator* allows fitting and increases the accuracy of NMR parameters calculated by DFT, and this approach resulted in better fitting with the HMCMC procedure. Using simulation objects allows the fitting of molecular properties (shifts) rather than direct line shapes (spectra). This approach introduces flexibility in the source of the NMR spectra, enabling the use of different spectrometer field strengths or spectral widths.

### Spectral Information Content

To simplify and accelerate the spectral fitting procedure, we considered the spectrum as smaller spectral intervals and fit these intervals rather than the whole spectrum. Dividing a spectrum into smaller regions of interest is similar to how an expert manually analyzes a spectrum and affords the workflow multiple benefits. The major benefit is that better resolved regions are analyzed first, allowing candidate compounds to be removed from consideration before assessing more congested regions of the spectrum.

To quantify the information content of spectral intervals, we use a concept from information theory in which the information content is quantified as information entropy, *H*, defined as
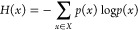
13

Here, our spectral space, *X*, is composed of possible compounds present within an interval that is readily available from the DFT-generated candidate library. The probability, *p*(*x*), of identifying a candidate compound in a region of an NMR spectrum is defined as the ratio of compounds predicted to appear in that interval to the total number of compounds in the candidate library. Intervals are then ranked by their entropy to determine the order in which these intervals are fit, with high-information entropy intervals fit first. In situations in which multiple intervals have the same entropy, the coincident intervals are ordered from least shielded to most shielded, as is a common strategy when analyzing spectra manually.

## Results and Discussion

### Performance Benchmarking

To evaluate the performance of the HMCMC fitting portion of the workflow, we conducted a series of benchmark tests. These benchmarks are designed to evaluate the ability of the HMCMC procedure to analyze the NMR spectra of reaction mixtures and autonomously identify the constituents. The value of our method is that it is tolerant of errors in the chemical shifts of the resonances in a spectrum of a crude reaction mixture, allowing us to use calculated spectra in place of those derived from experimental data. To test the impact of errors in the predicted chemical shifts of the reaction components, we considered three levels of NMR predictions with varying accuracy. The first, a rule-based NMR prediction implemented in ChemDraw, is characterized by a standard deviation of roughly 0.4 ppm in the predicted chemical shifts.^56^ The standard deviation of chemical shifts calculated by DFT using the functional and basis sets described above was found to be roughly 0.1 ppm. Finally, these approaches to chemical shift estimation are compared against the use of known spectra from a chemical library repository, which represents the best-case scenario of having known NMR spectra. Because our method requires a standard deviation parameter, we assign a chemical shift standard deviation of 0.01 ppm for molecules with a known spectrum. These three methods (ChemDraw, DFT, and library sources of NMR spectra) were compared in three benchmark tests: (1) fitting a complex spectrum containing n number of compounds using a candidate list of n molecular structures (in which n = 5 or 10); (2) the same procedure as (1) with the addition of random noise to the baseline of the true spectrum; and (3) fitting a complex spectrum containing 5 compounds using a candidate list of 10 molecular structures, with and without the addition of baseline noise.

**Figure 3:**
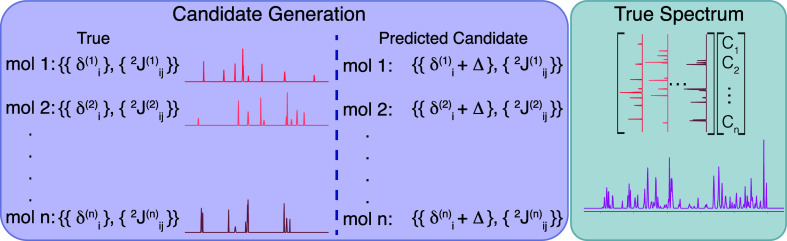
General procedure for creating the set of true and estimated candidate compounds in the benchmark testing. To generate a “True” library of spectra, each simulated compound is given a set of shifts and couplings which can be used to generate a spectrum. The simulated predictions for each compound are generated by adding noise, Δ, to the shifts. The noise is sampled from a normal distribution, with the variance determined by the level of theory used: Δ ∼ *N*(0,σ_Theory_^2^). The observed spectrum is generated by multiplying each library spectrum by a randomly sampled weighting parameter *C*_*i*_ and adding the spectra together.

To generate candidate compounds for these benchmark tests, a set of artificial NMR spectra was created, as illustrated in Figure 3. Each artificial spectrum was created by summing a series of component spectra, each of which was created by the following procedure: Each component spectrum contained 10 resonances with isotropic shifts selected from a spectral range of 0 to 10 ppm. In each component spectrum, each of the resonances was assigned a random integer intensity between 0 and 3. Of the ten resonances in each component spectrum, 5 were randomly selected to be coupled, with a *J*-coupling value of either 5 or 10 Hz. Each component spectrum was then assigned a weight, which represents the concentration of the compound in the mixture, by sampling from a Dirchlet distribution with a length parameter equal to the number of compounds in the spectrum. The spectra were then multiplied by their respective weights and stacked into a single composite spectrum, which mimics the experimental spectrum of a reaction mixture.

The test set for each simulation method (ChemDraw, DFT, and library) assessed the influence of error in the predicted isotropic shifts of the resonances in the component spectra on the ability to fit the component spectra to the composite spectrum. To simulate a case in which the candidate compounds are known but the predicted shifts have some error, each component spectrum from the artificial data set was copied, and the isotropic chemical shifts of each resonance were jittered. A point was sampled from a Gaussian distribution centered at 0 with a standard deviation dependent on the method of testing (σ ≈ 0.4 ppm for *ChemDraw*, 0.1 ppm for the DFT method herein, and 0.01 ppm for spectra reported in chemical libraries), and this point was added to the base isotropic shift value to introduce artificial error. The jittered component spectra were then fit to the composite spectrum to identify the weights of each component spectrum, despite the errors in the chemical shift. Each test was repeated 15 times, each time generating a new random set of spectra, and the results were averaged. The results for benchmarks 1 and 2 are summarized in Table 1, and benchmark 3 is summarized in Table 2.

**Table 1:** Summary of Results for Benchmark Experiments 1 and 2^a^

benchmark	N compounds	ChemDraw (MAE/%)	DFT (MAE/%)	library (MAE/%)
1	5	9	6	2
	10	6	5	2
2	5	7	5	2
	10	6	5	2

a In each benchmark, either 5 or 10 compounds are used to construct the spectrum, and the same 5 or 10 compounds are present in the candidate library. Benchmark 1 involves fitting the spectrum “as is”, while the spectrum in benchmark 2 includes baseline Gaussian noise. The MAE of the concentrations of the compounds is given for each test.

**Table 2:** Summary of Benchmark Testing for Benchmark 3^a^

	ChemDraw		DFT		library	
conditions	MAE/%	accuracy / %	MAE/%	accuracy / %	MAE/%	accuracy / %
no noise	9	64	5	79	1	97
noise	10	61	5	77	1	97

a In each test, 10 candidates are given in the library, of which only 5 are truly in the spectrum. One test is performed in which the spectrum is given as is, and a second test is performed by adding baseline Gaussian noise to the spectrum. The MAE in the concentrations of each candidate and the selection accuracy is given for each test.

#### Benchmark 1: Variable Ratios

With a sample size of 15 runs, the HMCMC procedure accurately predicted the relative concentrations of the compounds in a composite spectrum. The accuracy of the HMCMC assignment was proportional to the accuracy of the method used to simulate the NMR spectra, as evidenced by a monotonic decrease in MAE as the level of the simulated average error in chemical shifts decreased. Fitting the NMR spectra of 5 or 10 compounds to a simulated composite spectrum with average errors in chemical shifts consistent with predictions by *ChemDraw* (ca. 0.4 ppm) was accomplished with an MAE in the predicted weights of each component spectrum of 9 and 6%, respectively. The errors in chemical shifts representative of predictions by DFT (ca. 0.1 ppm) resulted in HMCMC fitting with MAE values of 6 and 5% for 5 and 10 components, respectively. Finally, the errors in chemical shifts representative of referencing known chemical shifts (ca. 0.01 ppm) led to an MAE of 2% for both tests. All methods are insensitive to the number of components in the composite spectrum, indicating that the HMCMC procedure can deconvolute even complex spectra with multiple overlapping peaks. In addition, the results suggest that NMR predictions that are computationally cheaper but less precise can be used in this context if the associated error in the predicted concentrations is acceptable. Therefore, the deconvolution of spectra with significant overlap or spectra with similar line shapes should be conducted with high-precision prediction methods, whereas the deconvolution of spectra with peaks that are well-spaced and easily identified can be conducted with less precise prediction methods.

#### Benchmark 2: Baseline Noise

In contrast to the artificial spectra used in our first test case, spectra obtained experimentally contain baseline noise due to both electronic noise and minor impurities arising from the solvent, reagents, substrates, or byproducts present in low concentrations. To test the ability of our approach to analyze NMR spectra in the presence of baseline noise, we conducted a benchmark test with noise purposefully included. To do so, a vector of random points was created such that the standard deviation of the vector was 0.01 (approximately 1% of the highest intensity peak in the spectrum), and this vector was added to the artificial spectrum used in Benchmark 1.

No significant difference was observed between Benchmark 1, which was conducted without baseline noise, and Benchmark 2, which included artificial baseline noise (Table 1). When the level of random error in the chemical shifts is consistent with the NMR spectra generated from ChemDraw, the MAE in the concentration of each component in the spectrum was 7 and 6% for spectra corresponding to 5 or 10 components, respectively. Using a level of random chemical shift error that is consistent with the errors in NMR spectra generated from DFT resulted in an MAE of 5% for both 5 and 10 components, and using a level of error consistent with chemical shifts obtained from known compounds resulted in an MAE of 2% for both 5 and 10 components. These results indicate that the accuracy of our approach does not suffer from the presence of baseline noise when predicting the weights of component spectra, demonstrating the ability of our workflow to deconvolute and analyze experimental spectra.

#### Benchmark 3: Missing Components

While it is important for an analytical approach to identify the relative concentrations of components in a reaction mixture correctly, it must also determine the identity of the components from a set of potential species in the mixture. Thus, a final benchmark test was designed to assess the ability of the HMCMC procedure to determine the identity and relative concentrations of compounds in the mixture corresponding to the composite spectrum. For this benchmark, components predicted to constitute less than 5% of the mixture were considered to be absent. This test was performed both with and without baseline noise added, as described in Benchmark 2.

For a sample size of 15 trials, following the procedure described above for HMCMC fitting, increasing errors in chemical shift once again led to a monotonic increase in MAE in the predicted component concentrations, as seen in Benchmark 1 (Table 2). The addition of baseline noise did not result in a larger MAE, as seen in Benchmark 2. For the tests with and without noise, chemical shift errors representative of predictions by *ChemDraw* (*ca.* 0.4 ppm) resulted in classifying each compound as present or absent in the reaction mixture with accuracies of 64 and 61%, and MAEs of 9 and 10%, respectively. Chemical shift errors representative of predictions by DFT resulted in accuracies of 79 and 77%, and MAEs of 5% for both tests. Chemical shift errors representative of referencing known compounds found in a library of chemical shifts led to accuracies of 97% and MAEs of 1% for both tests. These results indicate that our approach can identify the individual component spectra in a composite spectrum even when more candidate structures are provided than exist in the composite spectrum.

Inspection of the HMCMC analysis reveals that higher accuracy can be reached by varying the cutoff value. In cases in which the chemical shift error corresponds to that of literature-reported spectra, wherein the predicted chemical shifts are within *ca.* 0.01 ppm of the experimental chemical shifts, the HMCMC method falsely classified reaction components as absent when the true concentration was close to the cutoff value. For example, when the cutoff value was set to 5%, a compound with a true weight of 6% but a predicted weight of 4% was incorrectly classified as absent, despite the low absolute error in the predicted concentration. In practice, one may prevent this systematic error by adjusting the cutoff criteria to be more appropriate for the level of chemical shift error in the predicted component spectra. An alternative approach would be to subtract the spectra of the compounds that were initially confirmed to be present in the mixture and fit the remaining candidates to the residual.

### Experimental Testing

Having validated our HMCMC workflow, we tested the automated analysis of experimental data by analyzing several NMR spectra of crude reaction mixtures. During benchmarking, a tacit assumption was made that the position, intensity, and splitting pattern of the peaks in each component spectrum were not correlated. In this case, the probability of peak overlap is lower, and errors in predicted chemical shift are well tolerated, as indicated by the similar MAEs for simulated chemical shift errors between 0.01 and 0.4 ppm. However, the spectra of unique but structurally similar compounds can have similar NMR parameters and splitting patterns. To determine the applicability of our workflow to experimental data, we tested it on crude experimental spectra derived from real reaction mixtures. We considered the most difficult test and most useful application of this technology to be the analysis of reaction selectivity. The reactions presented herein were chosen because they form a mixture of products with similar structures and thus pose a difficult challenge for analysis. These examples evaluate the performance of the model in its intended applications, such as the analysis of the selectivity of reactions that can form one or more constitutional isomers.

#### Wittig Olefination

**Figure 4:**
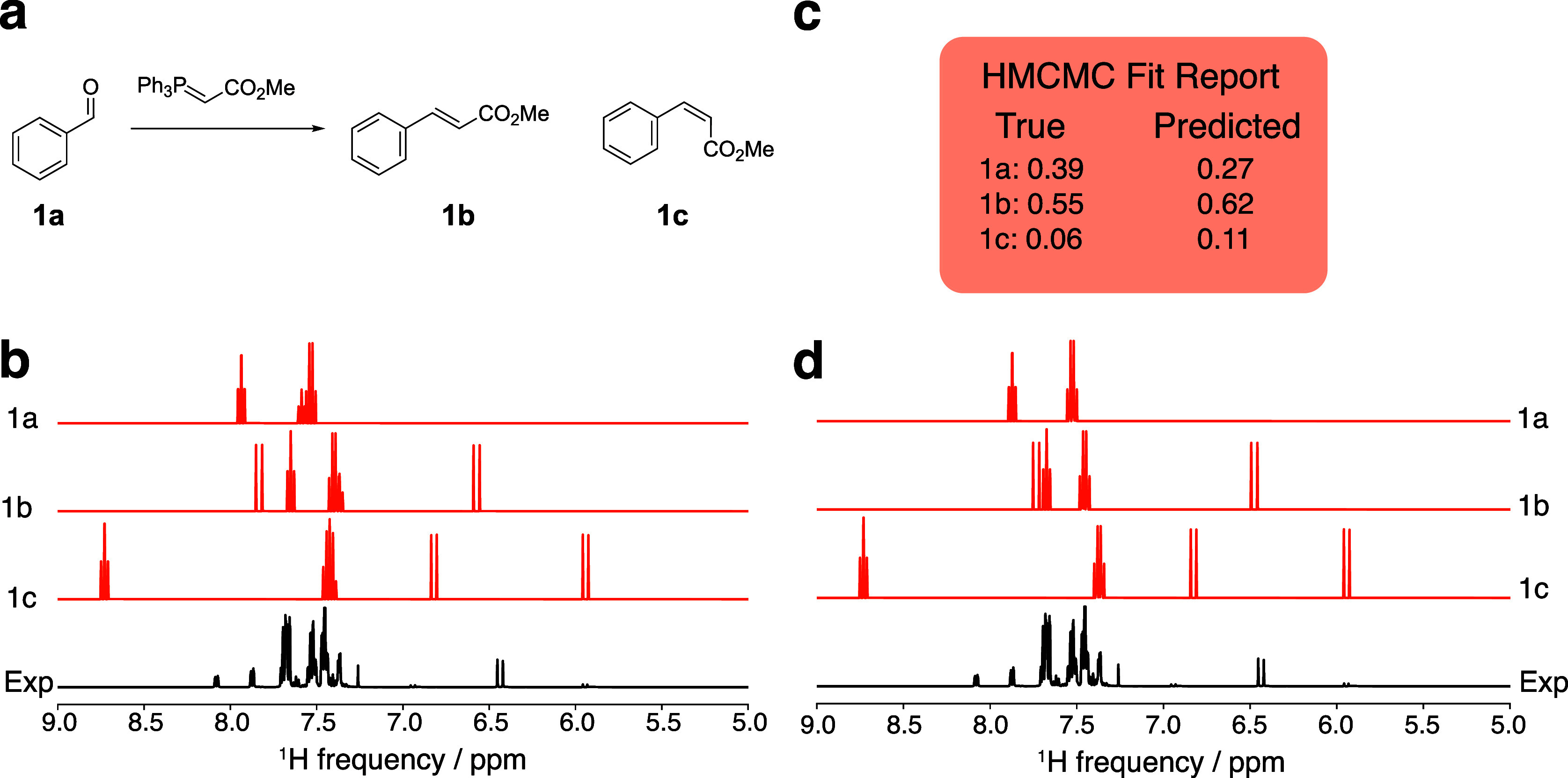
Results of HMCMC deconvolution of the spectrum from the Wittig olefination. (a) Candidate library for the Wittig olefination reaction, (b) initial DFT-generated spectra before HMCMC fitting, (c) final HMCMC fit report showing the predicted composition, and (d) final deconvoluted spectra showing the HMCMC-fit spectra of the molecules determined to be in the experimental mixture. The experimental spectrum was acquired at 500 MHz.

The first example we consider is a reaction that forms a mixture of olefin isomers, as shown in Figure 4. The Wittig olefination of benzaldehyde (**1a**) forms a mixture of E (**1b**) and Z (**1c**) olefin isomers, resulting in a simple candidate library of three compounds (two possible products and one starting material). The NMR parameters for all candidate compounds were calculated and calibrated as described above to yield a set of predicted NMR spectra (Figure 4b).

From the first iteration of the deconvolution procedure, all compounds in the candidate library were predicted to be present in the NMR spectrum in greater proportion than the threshold value of 5%. The HMCMC fitting of these spectra resulted in predicted relative concentrations that closely match the true values (Figure 4c). The model predicted the composition of the Wittig mixture with 100% classification accuracy (i.e., classifying each potential product as present or absent) and identified the concentrations of the present compounds with an 8% MAE, demonstrating an excellent ability to determine relative concentrations of reaction components. The deconvoluted spectra are shown in Figure 4d. The deconvolution procedure accurately predicts the relative ratios of components, despite the presence of individual peaks that are poorly aligned with the experimental spectrum, such as the peak near 8.7 ppm in compound **1c**. The chemical shift of this peak was poorly predicted by DFT and is characterized by a wide distribution of HMCMC-predicted chemical shifts. However, the chemical shifts of the other peaks corresponding to this molecule were fit with high accuracy, and so the overall fit of the spectrum is sufficient. Poor fitting may be alleviated by using more accurate DFT modeling techniques or by widening the bounds in the truncated Gaussian used to estimate chemical shifts.

This example further highlights a key aspect of fitting spectra via HMCMC: in regions where spectral peaks heavily overlap, the chemical shift predictions become less precise. This imprecision in the chemical shift fitting primarily results from the broadening step. As described above, broadening the spectrum increases the gradient information used by the fitting algorithm. The broadening, however, widens the line shapes and results in a loss of resolution in the sharp peaks and splitting patterns, which reduces the sensitivity to precise chemical shift positions. It is worth noting, however, that even with this broadening step, the fitting process can provide accurate predictions of relative concentrations, as long as the chemical shifts are approximately in the correct positions.

#### Arene Borylation

**Figure 5:**
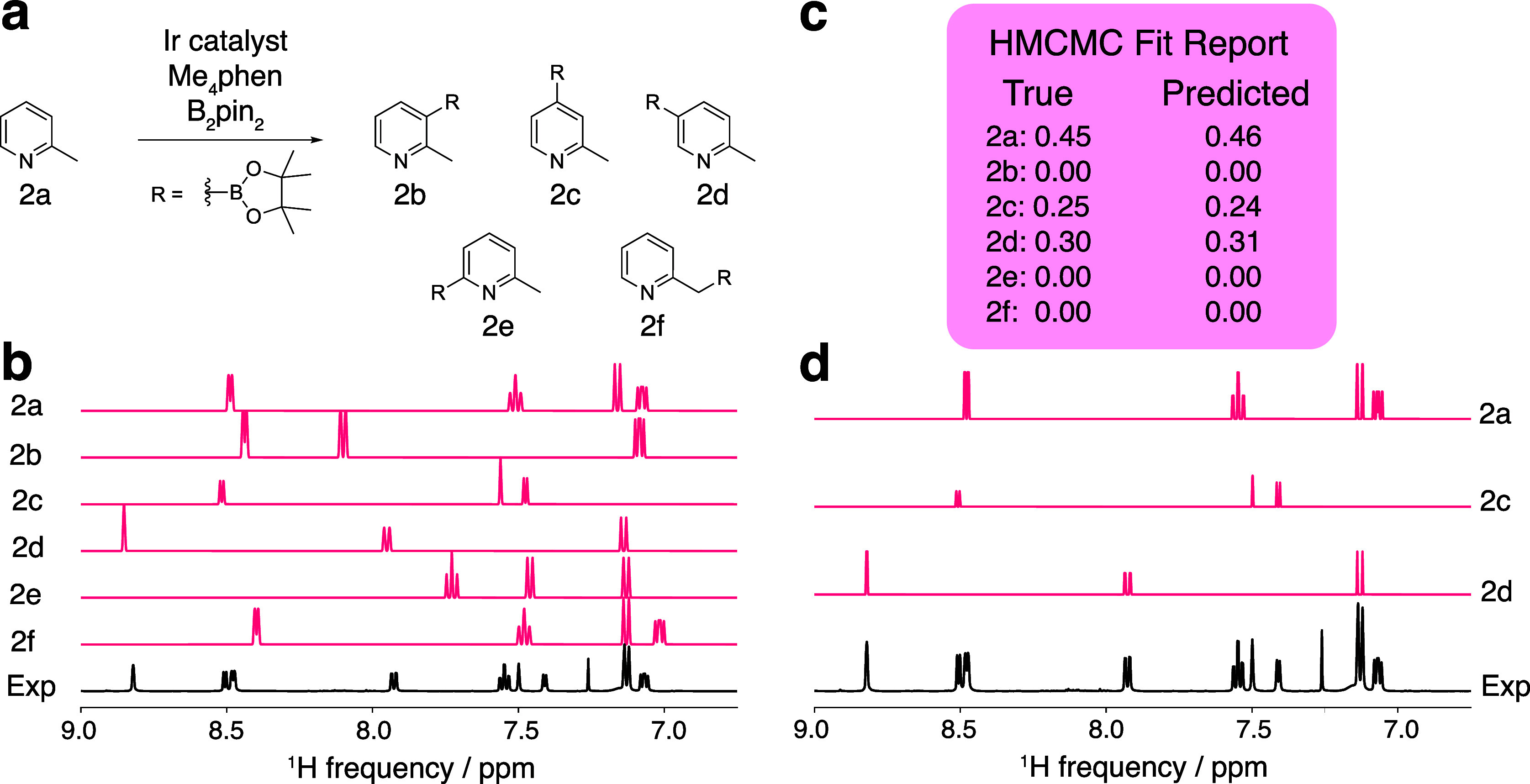
Results of HMCMC deconvolution of the spectrum from the picoline borylation. (a) Candidate library for the arene borylation reaction, (b) initial DFT-generated spectra before HMCMC fitting, (c) final HMCMC fit report showing the predicted composition, and (d) final deconvoluted spectra showing the HMCMC-fit spectra of the molecules determined to be in the experimental mixture. The experimental spectrum was acquired at 500 MHz.

In the next example, we analyzed the NMR spectra of the crude reaction mixture resulting from the borylation of C–H bonds of aromatic compounds (Figure 5). The borylation of picoline (**2a**) could occur at any of the arene C–H bonds (**2b**–**e**) or at the benzylic C–H bond (**2f**). Therefore, we presented the model with the 6 potential borylation products, as shown in Figure 5a. NMR parameters for all candidate compounds were calculated and calibrated as described above to yield a set of predicted NMR spectra (Figure 5b). Because the starting material is a known compound, an NMR spectrum of the starting material was obtained and fit using the *MRsimulator*, as described above, to obtain a better initial trial spectrum.

Following the first iteration of the deconvolution procedure, compounds **2b**, **2e**, and **2f** were removed because their predicted concentrations were all below the 5% concentration cutoff. A final iteration of the deconvolution procedure was performed with the remaining compounds **2a**, **2c**, and **2d** to improve the resulting fit. As shown in Figure 5c, the procedure, once again, predicted the presence or absence of candidate compounds with 100% accuracy and predicted the concentrations of each compound present with an MAE of 1%, demonstrating the ability of our method to determine which components are present in the spectrum of a crude reaction and the relative concentrations of the components. The deconvoluted spectra are shown in Figure 5d.

#### Borylation of Polyaromatic Compounds

**Figure 6:**
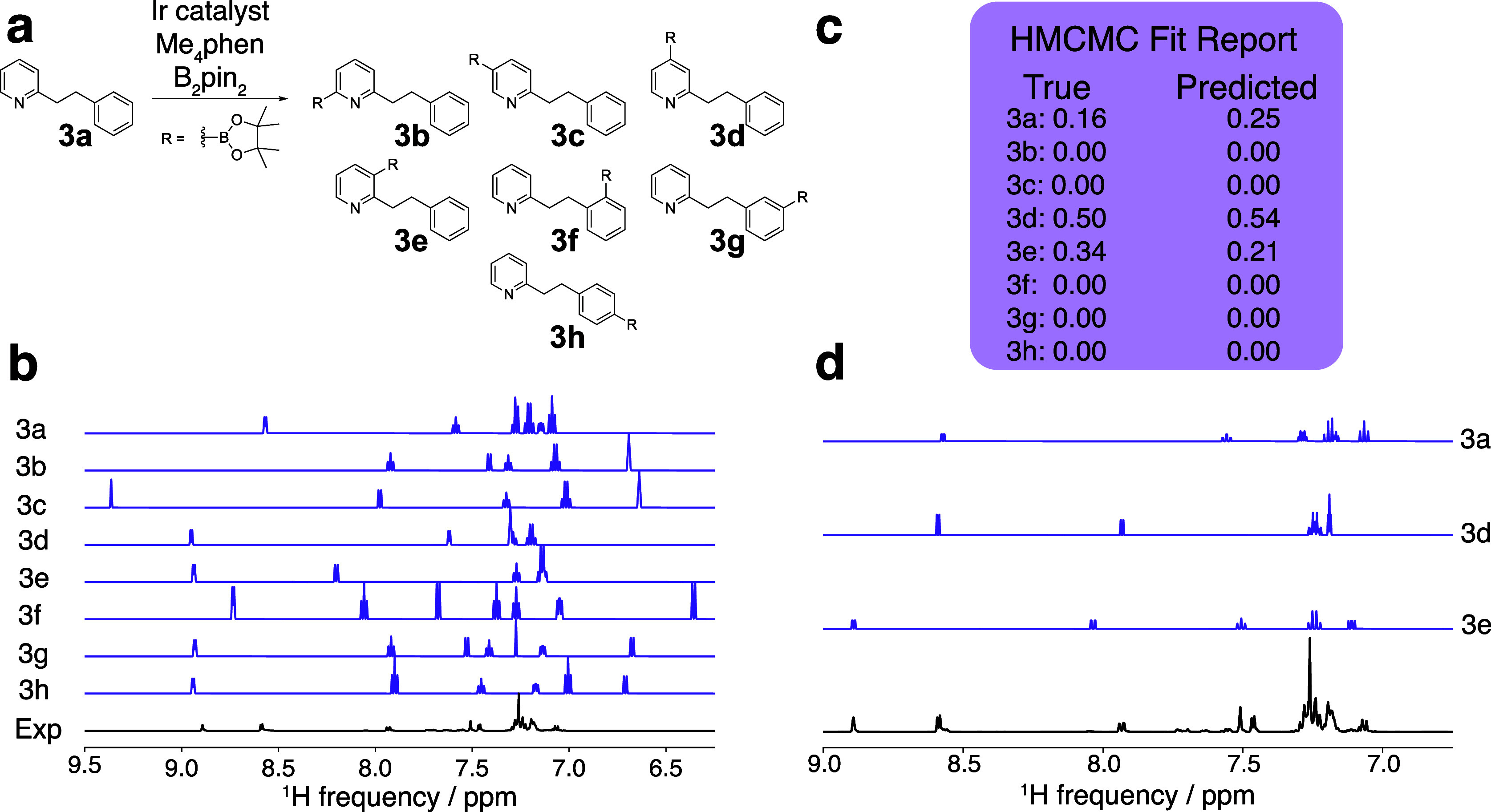
Results of HMCMC deconvolution of the spectrum from the 2-phenylethylpyridine borylation. (a) Candidate library for the multiarene borylation reaction, (b) initial DFT-generated spectra before HMCMC fitting, (c) final HMCMC fit report showing the predicted composition, and (d) final deconvoluted spectra showing the HMCMC-fit spectra of the molecules determined to be in the experimental mixture. The experimental spectrum was acquired at 500 MHz.

We next considered a significantly more difficult example: the borylation of 2-phenylethylpyridine (**3a**, Figure 6). The number of potential products from this reaction is larger, and many of the resonances of the protons in these products overlap. In addition, the experimental spectrum contains unassigned, low-intensity signals that correspond to the presence of impurities in the reactant. The borylation can occur at any aromatic C–H bond of 2-phenyethylpyridine. Borylation of secondary benzylic C–H bonds is unlikely; therefore, products from reactions at those positions were not considered. In total, the candidate library consisted of the 8 compounds shown in Figure 6a. To compensate for the impurity peaks, a cutoff of 10% was used during the HMCMC fitting. The NMR parameters for all candidate compounds were calculated and calibrated as described above to yield a set of predicted NMR spectra (Figure 6b). Because the starting material was available, the NMR spectrum of the starting material was acquired, and the chemical shifts and splitting patterns of the peaks in the experimental spectrum were fit using the *MRsimulator*, as described above, to obtain a better initial trial spectrum.

The HMCMC procedure is a sampling method, and one of the sampled variables is the chemical shift. Therefore, the distribution of HMCMC-predicted chemical shifts is indicative of how well the individual spectra fit to the experimental spectra. For example, a successful fitting procedure will result in a narrow distribution of chemical shifts, and an unsuccessful procedure will result in a wide distribution of chemical shifts with large standard deviations. Because the HMCMC procedure fits a DFT-predicted spectrum to an experimentally obtained spectrum, a large standard deviation indicates inaccurate predictions of chemical shifts by DFT or highly overlapping regions where peak assignment may be ambiguous.

After the first iteration of the deconvolution procedure, compounds **3b**, **3c**, **3f**, and **3h** were removed from the set of potential products because their predicted concentrations were below the 10% cutoff. For each of the remaining compounds (**3a**, **3d**, **3e**, and **3g**), the majority of the predicted chemical shifts deviated little from the true value (approx < 0.1 ppm). However, some resonances were characterized by large standard deviations in the HMCMC-predicted chemical shift. A large standard deviation in the predicted chemical shifts occurs when peaks in the trial spectrum cannot be fit to the experimental spectrum, either because the compound does not exist in the reaction mixture or because the initial trial shifts were very poorly predicted by DFT. To address this issue, in the second HMCMC iteration, the standard deviation of the Gaussian distributions used for the chemical shifts was set to three standard deviations of the DFT error (0.3 ppm), and the HMCMC procedure was repeated. After this iteration, compound **3g** was determined to be absent, and only **3a**, **3d**, and **3e** remained. The standard deviations in the chemical shift predictions for **3a**, **3d**, and **3e** were small, and a final application of the deconvolution procedure was performed to improve the fit. As shown in Figure 6c, the procedure yielded a prediction accuracy of 100% and an MAE of 9% in the concentrations of components, demonstrating that our approach can deconvolute the NMR spectra that contain extensive overlapping of the resonances.

#### Oxidation of Heptane

Finally, we considered a complex mixture simulating the nonselective oxidation of the C–H bonds in heptane to form alcohol, ketone, aldehyde, and carboxylic acid products. This example simulates an experimentally obtained spectrum by mixing commercially available compounds. This example poses a stringent test of the procedure because the spectra of many of the compounds are similar to each other. Thus, most of the peaks in the spectrum of the mixture overlap.

To generate a list of candidate compounds for this simulated reaction, we considered all probable products of the oxidation of heptane (**4a**): compounds with a hydroxyl group at each nondegenerate carbon atom, ketones at each nondegenerate carbon, and aldehyde and carboxylic acid groups at the terminal carbons (**4b**–**j**). In total, the candidate library comprised 10 compounds. Three unique regions of the spectrum were identified by considering their spectral information, as described in the Methods above. These regions were used sequentially to deconvolve the experimental spectrum.

**Figure 7:**
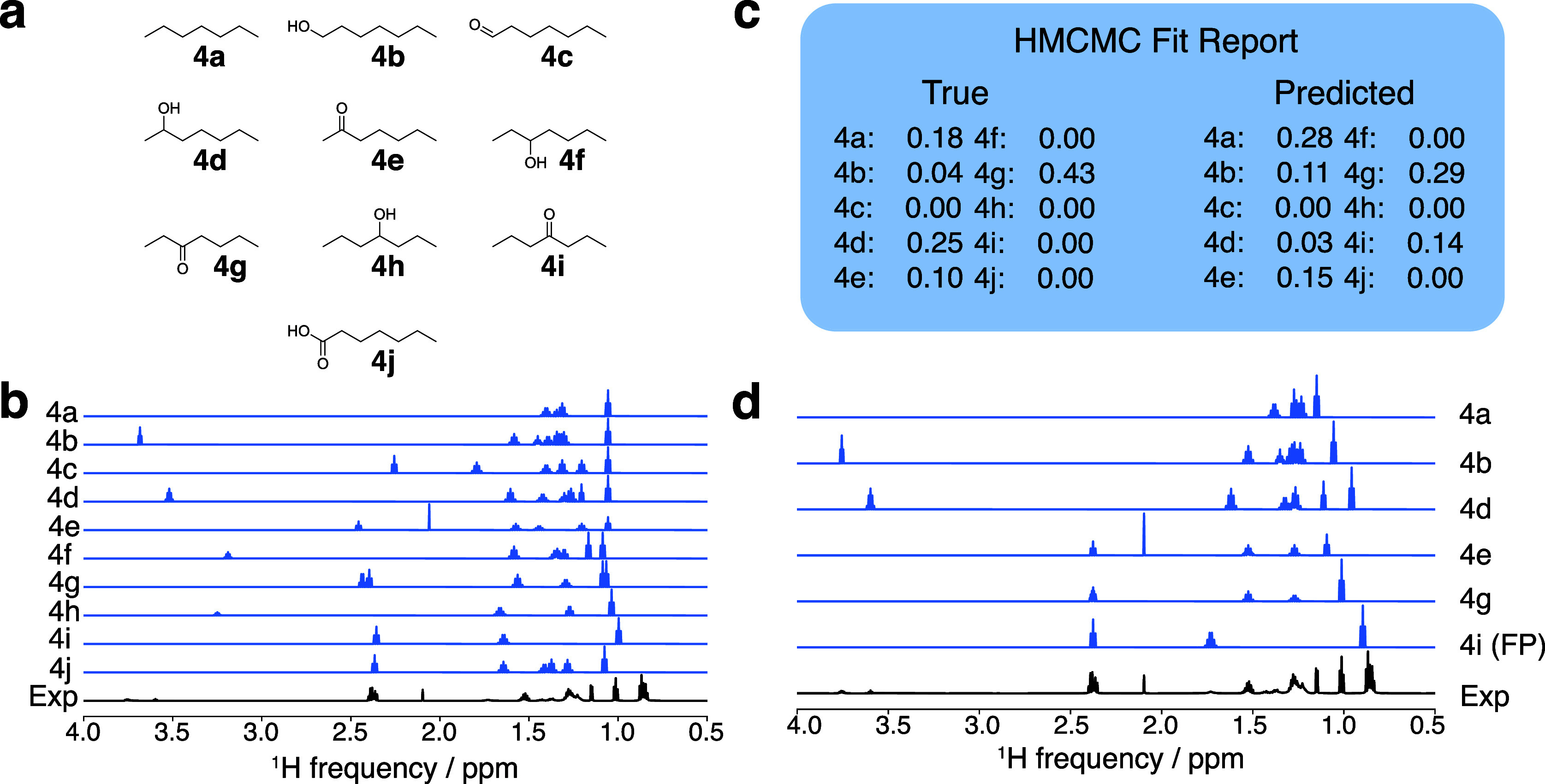
Results of HMCMC deconvolution of the spectrum from the heptane oxidation simulation. (a) Candidate library containing compounds **4a**–**4j**, (b) initial DFT-generated spectra before HMCMC fitting, (c) final HMCMC fit report showing the predicted composition, and (d) final deconvoluted spectra showing the HMCMC-fit spectra of the molecules determined to be in the experimental mixture. The false positive prediction is denoted as (FP). The experimental spectrum was acquired at 700 MHz.

The first region of interest identified by the spectral information content procedure was the region with a chemical shift greater than 8 ppm. Analysis of this region revealed whether heptanal or heptanoic acid (**4c** and **4j**) was present in the spectrum. In the first iteration of the HMCMC procedure, both compounds **4c** and **4j** were excluded. Next, the information-dense region of the spectrum between 3 and 4 ppm was analyzed. In the library of candidate spectra, all peaks in this region corresponded to a methine proton α to a hydroxyl group. Thus, the presence or absence of peaks in this region revealed the presence or absence of 1-heptanol (**4b**), 2-heptanol (**4d**), 3-heptanol (**4f**), and 4-heptanol (**4h**). In an iteration of the HMCMC procedure, compounds **4f** and **4h** were removed from the list of components. Analysis of the region of the spectrum from 0 to 3 ppm left **4a**, **4b**, **4d**, **4e**, **4g**, and **4i** in the candidate library. After an iteration of the HMCMC procedure, none of the compounds were removed, and as shown in Figure 7c, the procedure yielded a prediction accuracy of 90%, and the relative concentrations were determined to be [0.28, 0.11, 0.03, 0.15, 0.29, and 0.14]. These concentrations can be compared to the true concentrations of [0.18, 0.04, 0.25, 0.10, 0.43, and 0.00], for an MAE of 16%. Despite compound **4b** falling below the 5% cutoff criteria, its presence was established from analysis of the midfield region (3–4 ppm), and it was retained in the library.

Analysis of the modest performance of the HMCMC procedure when analyzing this spectrum shows that it was difficult to fit accurately, and some peaks and concentrations were assigned incorrectly. The likely reason for the imprecision is the large extent of overlap between spectra and the lack of functional groups that allow for unambiguous assignment. The chemical shifts and splitting patterns of the methyl and methylene protons of **4a**–**j** are similar, creating a multimodal optimization surface which is difficult to deconvolute. Indeed, the deconvolution of this spectrum by experts is also challenging.

To address this difficulty, future efforts can introduce ^1^H–^13^C HSQC or ^13^C spectra to provide additional information that allows for more accurate deconvolution of ^1^H NMR spectra because the additional information from the carbon spectrum will aid in excluding compounds, and the signals in ^13^C NMR spectra overlap less than those in ^1^H NMR spectra due to the greater dispersion of chemical shifts. This approach can be coupled with expanding the standard deviation for the initial distribution of chemical shifts used for the analysis of ^1^H NMR spectra. This expansion could lead to better deconvolution. Importantly, the workflow described herein can be applied to ^13^C NMR prediction with minor modifications, enabling further method development.

## Conclusions

In conclusion, we have developed an automated workflow for the identification and quantification of novel chemical compounds in a reaction mixture. We demonstrate that this approach can be used to deconvolute and analyze experimentally obtained crude NMR spectra that contain multiple isomeric products. We present this workflow as a series of modules so that practitioners can adapt it further to their needs. The method can enable researchers to automate the analysis of HTE campaigns that generate large numbers of previously undescribed compounds. This contribution may transform our ability to generate training data for machine learning models and assist drug discovery campaigns during diversity-oriented synthesis.

## Data Availability

The code referenced in this work is deposited and available as a notebook for download at https://github.com/mVenetos97/nmrmix. The NMR spectra referenced in this work are available on the aforementioned Github Repo.
